# Deep neural architecture for natural language image synthesis for Tamil text using BASEGAN and hybrid super resolution GAN (HSRGAN)

**DOI:** 10.1038/s41598-023-41484-9

**Published:** 2023-09-02

**Authors:** M. Diviya, A. Karmel

**Affiliations:** 1 School of Computer Science and Engineering, Vellore Institute of Technology, Chennai, Tamilnadu 600127 India; 2 School of Computer Science and Engineering, Vellore Institute of Technology, Chennai, Tamilnadu 600127 India

**Keywords:** Engineering, Computational science, Computer science

## Abstract

Tamil is a language that has the most extended history and is a conventional language of India. It has antique origins and a distinct tradition. A study reveals that at the beginning of the twenty-first century, more than 66 million people spoke Tamil. In the present time, image synthesis from text emerged as a promising advancement in computer vision applications. The research work done so far in intelligent systems is trained in universal language but still has not achieved the desired development level in regional languages. Regional languages have a greater scope for developing applications and will enhance more research areas to be explored, ruling out the barrier. The current work using Auto Encoders failed at the point of providing vivid information along with essential descriptions of the synthesised images. The work aims to generate embedding vectors using a language model headed by image synthesis using GAN (Generative Adversarial Network) architecture. The proposed method is divided into two stages: designing a language model TBERTBASECASE model for generating embedding vectors. Synthesising images using Generative Adversarial Network called BASEGAN, the resolution has been improved through two-stage architecture named HYBRID SUPER RESOLUTION GAN. The work uses Oxford-102 and CUB-200 datasets. The framework efficiency has been measured using F1 Score, Fréchet inception distance (FID), and Inception Score (IS). Language and image synthesis architecture proposed can bridge the gap between the research ideas in regional languages.

## Introduction

Image synthesis from natural language descriptions is a field of research focusing on generating visual content, such as images or illustrations, based on textual descriptions or prompts. It involves training machine learning models, typically deep learning techniques, to understand the connection between text and corresponding visual output. Text-driven image synthesis aims to develop a system that can generate meaningful and accurate images based on text features. Various applications include creative Design generation of visual content for various design purposes, such as illustrations for books, magazines, or websites.

Virtual Worlds and Gaming: It can automatically generate visual assets, such as landscapes, characters, or objects, in virtual worlds or video games based on textual descriptions or procedural generation. Data Augmentation for creating synthetic images for training machine learning models in computer vision tasks, helping to expand the size and diversity of available training data. Storytelling and Visualization: It can aid in the generation of visual representations for storytelling, helping to illustrate scenes or concepts described in written narratives.

Text-to-image synthesis typically involves training a variational autoencoder (VAE) or a generative adversarial network (GAN) on a large dataset of paired text and corresponding images. The model understands to map the text to a visual feature by capturing the underlying patterns and relationships in the training data. While significant progress has prompted Visual content generation from text, highly detailed and realistic images that accurately capture the nuances of textual descriptions remain challenging. However, recent advancements in deep learning techniques, including larger models and improved training methodologies, have shown promising results in generating visually coherent and contextually relevant images from text prompts.

A unique text-based image stylization system. Several steps are unsupervised. Style images are initially divided into foreground and background images. Then, the primary foreground colour is gathered, and binarized spatial shapes like text, symbols, and icons are assigned to create content images. Picture style transfer applies the foreground picture’s style to the content image^1^. Researchers proposed a technique that stylizes shapes such as text, symbolic representations, and patterns in binary images using an input-style image. Then the styled geometric shape and backdrop image are combined. Legibility-preserving structure and texture transfer methods have been employed to reduce discrepancies between both images^2^. Followed with other architectures, the proposed model may draw the necessary outcomes for creating higher-quality image synthesis by synthesising from simple to complicated and easy to difficult^3^.

Generative Adversarial Networks (GANs), proposed by Ian Good Fellow, is an eminent idea that plays various roles in every computer vision application. It is an unsupervised model that acts as a generative model in synthesizing novel images from similar images, generating new tunes from the existing ones^4^. The model proposed by the researchers works better for natural scenes and faces. The work aims to bring out the robustness of GAN apart from its performance^5^. Yanhua Li et al. worked in synthesizing dressing images from the given input images, which is termed fitting GAN. The model learns the input images, and the output generated according to the fitting. The model used L1 regularization and adversarial loss function^6^. Jezia et al. created a model that helps synthesis realistic images from a story text using spatial relations of the words. Followed by using two-stage models of generator and discriminator, the low-resolution images are converted to high-resolution images, and a KNN is used to compare the image quality score^7^. Valuable progress has been made in computer vision in image synthesis applications. The researchers concentrated on preserving the semantic information of the images. Super-resolution GAN is used for enhancing the resolution and quality of low-resolution images. It is advantageous to upscale an image without losing too much detail or introducing artifacts. The basic idea behind a super-resolution GAN is to train a generator network to produce improved-resolution images from low-resolution counterparts.

BERT (Bidirectional Encoder Representations from Transformers) is a well-trained language model. Transformer architecture is the basic block and has overtaken the other natural language processing tools. It is trained using massive corpus of text data and can understand the context and meaning of words and sentences. The critical feature of BERT is its bidirectional training, considering contexts at both ends of a word during training. This allows us to capture deeper semantic understanding and handle tasks that require understanding the closeness across different sentence parts. The model has been trained on large amounts of text from means like books, articles, and websites, which enables it to learn a broad range of language patterns and nuances.

After pre-training, BERT can be further trained on task-specific datasets with minimal adjustments to the model architecture. This fine-tuning process allows us to adapt to specific tasks and improve performance. It has paved the way for advancements in NLP. It has inspired the development of several other transformer-based models, such as GPT-3, RoBERTa, and ALBERT. These have achieved remarkable results in a comprehensive understanding of language and generation tasks.

The hands of language models have acquired various fields of applications. The researchers worked on BERT model for news classification with enormous data combined with spark. The performance of the BERT proves to be outstanding even when we have extensive data under study^8^. In NMT, BERT works better as contextual Embedding than fine-tuning for downstream language understanding tasks. This inspires us to improve BERT’s NMT use. The BERT-fused model first uses BERT to uproot depictions for an input sequence, then uses attention mechanisms to fuse the representations with every layer of encoder and decoder of the NMT model^9,10^.

## Related works

Many applications include various language models. The researchers proposed a language model using relational features where the text is represented as feature types including the n-grams of words, Character n-grams, etc., The method analyses the distance between the available tokens across the document. It concentrates on binary features, whereas the model does not consider longer-range relation features. The second approach followed was by using keyword detection and vectorization of the corresponding keywords words^11^. Various field works have been attempted in various applications for languages. However, the researchers developed a pre-trained model in extractive summarization for the biomedical domain, which is a challenging approach. They trained the sentence using the BIOBERTSUM language model, which follows the sentence position embedding mechanism^12^. While working with the embedding mechanism, the model taken in hand by the researchers is a unified model that can identify multiple paraphrases in an input sentence pair. The preprocessing method generates sentence embedding vectors using the sentence-BERT model^13^. Many researchers proposed multiple streams of methodologies in processing text input to arrive at better information for further processing. The survey done by the authors in lining up various word representation models starting from raw text into meaningful vectors includes preprocessing by tokenization, noise removal, segmenting words followed by stemming, and lemmatization ends up with pos tagging^14^. Even though applications may vary, the primary task that involves text input records needs an understanding of how statistical properties of the sentence or word are analyzed. They have incorporated methodologies such as ensemble models instead of Continuous Bags of Words, Skip-Gram, etc.^15^.

The publication of BERT and, more subsequently, GTP-3 represented a significant advancement for NLP. They provided an overview of the most significant language representation learning models developed for NLP and discussed the development of these models over time. In addition, it provides a summary, a comparison, and a contrast of these various models on sentiment analysis, then proceeds to evaluate their primary benefits and drawbacks^16^. Researchers proposed two parameter-reduction methods to reduce BERT memory usage and training speed. The methods scale better than BERT. A self-supervised loss that models inter-sentence coherence reliably improves preliminary tasks with multi-sentence inputs. Top model achieves state-of-the-art scores on the GLUE, RACE, and \squad benchmarks with less parameters than BERT-large^17^

BERT is an all-purpose language representation model that allows computers to use the rich, two-way context contained in natural language texts. The sequence-transduction model transformer is used for the attention mechanism. In the approach, classification is carried out in parallel for each word in the input word sequence to determine whether each word can function as an antecedent. The machine-readable text requires a language model. A linguistic model can predict the likelihood of a context-related term. Most language models in research are based on unidirectional training, which seems daunting. BERT is bidirectional, unlike Elmo. Transformer encoder-based word Embedding is used^18^. A fine-tune and classify patents with the model. The technique outperforms CNN with word embeddings on over two million patent datasets. They concentrated on patent claims alone. Indicating that patent claims alone can yield state-of-the-art classification results, contrary to popular belief^19^.

They fine-tun the pre-trained BERT with much text, yielding rich text information for image production. Position Embeddings (PEs) have been proposed to reflect word order in Transformer-based systems like BERT. No formal framework exists to investigate these empirically-driven, high-performing models. Translation invariance, monotonicity, and symmetry of PEs capture word distance in vector space. These features formalise PE behavior and enable principled sinusoidal PE reinterpretation. The proposed work offers a new probing test, “identical word probing,” and indicators using mathematical functions to objectively detect general attention patterns related to the qualities described above^20^.

Multi-perspective fusion was introduced to improve image synthesis. The generator uses a dynamic selection approach to match text and image features. In contrast, the discriminator uses a multi-class discriminant method using mask segmentation images as the different types to improve discrimination^21^. The joint probability of image tokens and their related layout tokens are observed using a joint-decoding transformer which gives extra observed data to describe complicated scenes—added with Layout-Vqgan invested in encoding and decoding extra information concerning complicated scenarios.

Attention approach to object generator may provide fine-grained features on targeted objects using an effective dual generator^22^. When the initial images are not properly formed, the fuzzy image contents can be refined with the help of a dynamic memory module introduced by the proposed method. The researchers developed a novel approach called multi-perspective fusion to improve text-to-image synthesis. In this approach, the generator incorporates a dynamic selection mechanism to match text features with image features, enabling more accurate synthesis. Meanwhile, the discriminator utilizes a multi-class discriminant method, where mask segmentation is introduced as an additional type to enhance its discrimination capacity^23^. The proposed framework, called RaSeedGAN (RAndomly-SEEDed super-resolution GAN), is designed to evaluate field quantities from randomly sparse sensors without relying on full-field high-resolution training. By utilizing random sampling, the algorithm gains fragmentary perspectives of the high-resolution underlying distributions. Even when sparse or noisy, the findings are promising^24^. The authors considered methodologies for generating statistical properties of the text. On the other hand, synthesizing the corresponding images for text input is challenging^25^. Synthesising high-quality images for computer vision applications is a challenging task. To overcome the disadvantage of a single-stage GAN network, the authors brought a two-stage network, resulting in an enhanced resolution image^26–28^. This work proposes keyframes selection strategy for video description using a boundary-based method that allows the system to encode visual information by picking a small subset of keyframes and construct a video description without considerable degradation^29^.

## Text-to-image synthesis model

**Figure 1:**
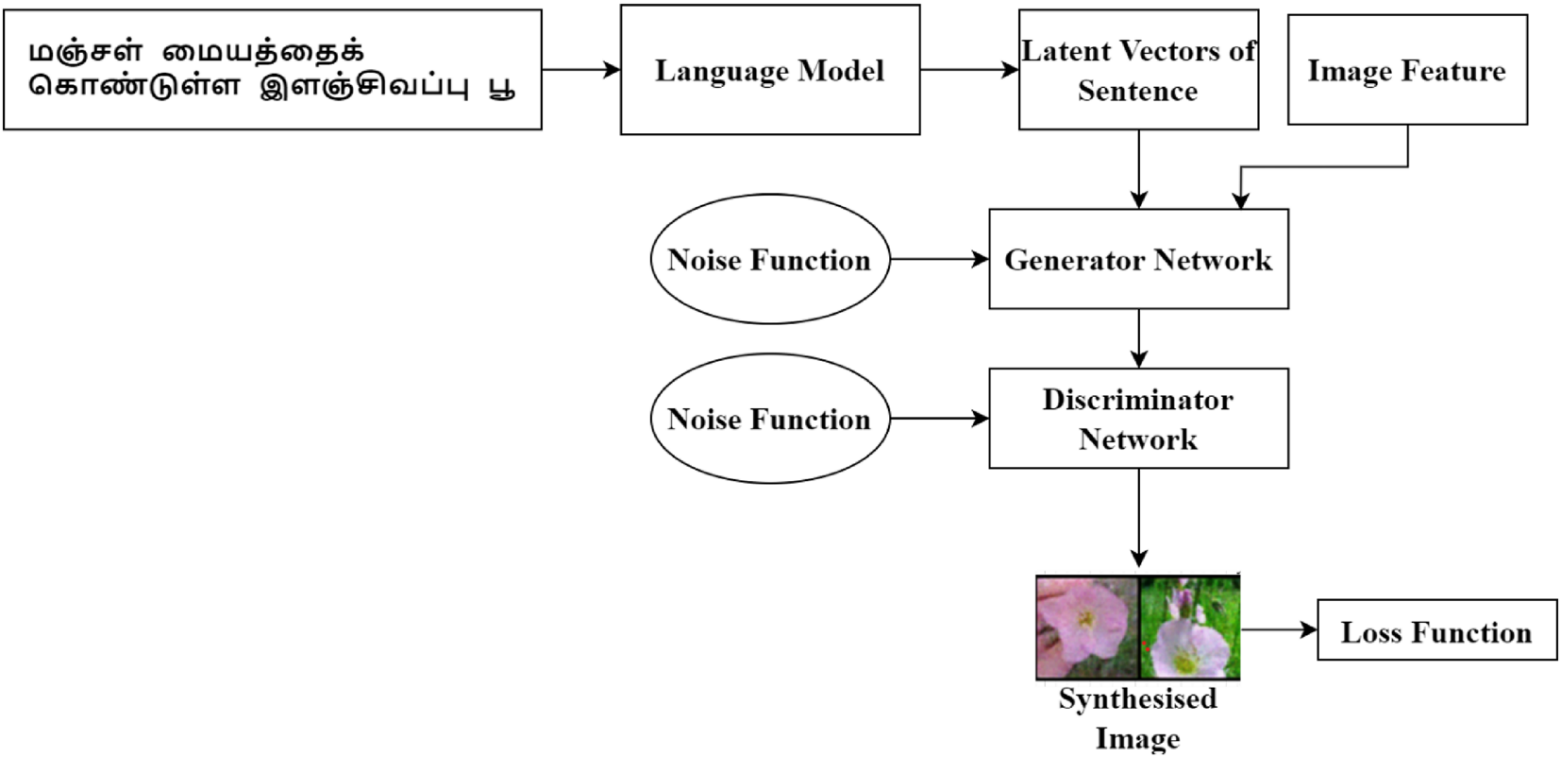
Text to Image Synthesis Architecture.

The architecture is designed for synthesizing images for corresponding Tamil text descriptions. The architecture proposed works well based on the statistical information. Since Tamil is a morphologically rich language, it is necessary to look into the language model and the synthesizer network. A new dataset was created by applying Google Translator to translate the English text of Caltech UCSD -Birds 200 and Oxford-102 datasets into the Tamil language. The image synthesis architecture is shown in Fig. 1.

The model leverages the ability of TBERTBASECASE to generate meaningful sentence embedding. The model is fused with TBERTBASECASE and BASEGAN by introducing language model word Embedding. The proposed method generates improved resolution over realistic images for Tamil text on two demanding datasets.

### Dataset

**Figure 2:**
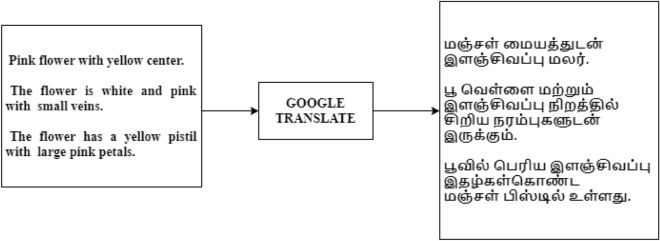
Text Translation using Google Translate.

The Oxford 102 flower dataset and CUB 200 text have been collected. Tamil sentence for the corresponding English text is translated using Google Translate to create the corpus and was trained. The Oxford-102 contains 102 classes, each with a set of image files and descriptions. Figure 1 shows how Google Translate translated English sentences into Tamil text files, probably about 3000 sentences. The CUB 200 dataset was also developed for experimentation. Figure 2 shows the translation.

### TBERT BASECASE model

The language model is a preliminary work that focuses on understanding the statistical properties of the input text, which results in the generation of text vectors. The BERT model is the initial transformer framework developed for processing text which is enhanced into various versions such as masked, unmasked, cased, and uncased, depending upon the corpus under learning and language taken for training. The proposed language model tried to use the BERT BASECASE model trained over Tamil text corpus by fine tuning the last layer of processing. The sentence’s feature study is done using a BERT named BERT BASECASE model. The proposed model is the TBERTBASECASE model, which is trained over Tamil input text and aims to study the properties of the sentence and generate corresponding text vectors. The BERT model is advantageous over other language models like GLOVE embedding, etc.^30,31^. Since the algorithm works bi-directional from left and right, it is unique in processing the text features^32^. The proposed TBERT BASE CASE model takes input text sentences followed by hidden layers. The TBERT BASECASE model has 12 layers which have 768 hidden units. It can process data with hyperparameters which are around 110 M. In Tamil the morphology of the language itself is a challenging task.

Token embeddings are the initial token representations which is feed in to BERT transformer blocks. Transformer blocks start with self-attention. It lets each token recognise and value all other tokens in the input sequence. Self-attention compares tokens and creates weighted sums of token embeddings to calculate attention weights. This method helps the model grasp local and global input sequence dependencies. After self-attention, the transformer block has residual connections. Residual connections let the model preserve token embedding information and avoid the vanishing gradient problem during training. Self-attention output is added element-by-element to token embeddings. A transformer block refines the tokens in the input sequence. Contextualized embeddings combine static token embeddings with contextual information from self-attention and the FFN. BERT has numerous transformer blocks stacked. The model captures more complicated relationships and refines token representations at each layer by passing the output of each block to the next block. BERT’s word embeddings are the last transformer block’s token representations.

**Figure 3:**
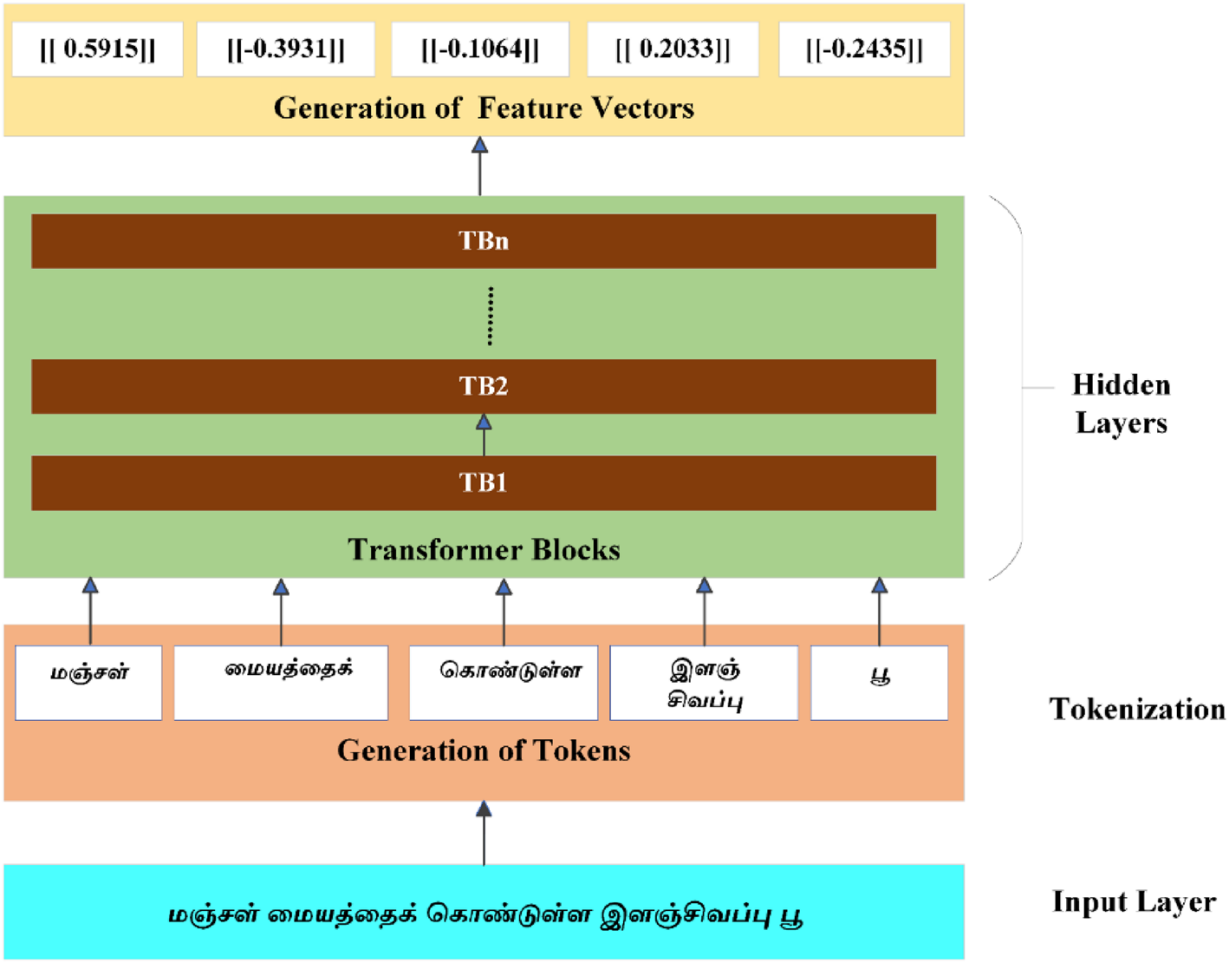
TBERTBASECASE language model.

The schematic representation of text processing using the TBERTBASECASE is presented in Fig. 3, and embedding vectors of the text are depicted in Table 1.

**Table 1:** Feature vector representation of the input text.

Tamil text	Tokens	Romanized pronunciation	Meaning	Text vector
		məɲdʒəɭ	Yellow	[[0.5915]]
		məjjət̪t̪əjk	Center	[[− 0.3931]]
		koɳɖʉɭɭə	Containing	[[− 0.1064]]
		ʲɪɭəɲdʒɪʋəppʉ	Pink	[[0.2033]]
		pu	Flower	[[− 0.2435]]

Consider the given set of sentences as t and the text token values from $[{t}_{1}{\dots t}_{n}]$ fed as input tokens to the input layer. The results pass through various hidden layers, which are customized according to the given Tamil input feature tokens and generate feature vectors ${[f}_{1},{f}_{2\dots }{f}_{n}]$ by employing concatenation or summing up the last layer vectors of the hidden layers of the transformer depicted as ${H}_{o}$. The objective function of the TBERT BASE CASE model is represented as,

1 $$\varphi \left( t \right) = t_{1} ,t_{2} , \ldots t_{n}$$

2 $$H_{o} \left( t \right) = H\left( {t_{1 } ),H(t_{2} } \right), \ldots H\left( {t_{n } } \right)$$

3 $$logT_{BERTBASECASE} \approx \mathop \sum \limits_{1}^{n } log\left( {H(t_{1} } \right)\left| {\left| \cdots \right|} \right|H(t_{n} ))$$

4 $$Embedding_{Text } = Elemetwise\,sum[T_{i} \ldots T_{n} \left] + \right[C_{0} \ldots C_{k} ]{ }$$

where n denotes the number of tokens in the given text, t is the Tokens of the given text, and Eq. (1) represents the various text tokens of the text. The concatenation of vectors in hidden layer is represented in Eq. (2). Equation (3) represents the objective function of the TBERT BASE CASE model. Each hidden layer consists of *T*_*i*_*…T*_*N*_*-*Token embeddings and C_*0*_*…C*_*k*_—contextualised embeddings and its Elementwise sum is drafted in Eq. (4).

### BASEGAN model for image generation

The Generator Discriminator network (GAN) proposed is a base image synthesis model deployed using Tamil text-to-image synthesis network. Earlier, many image synthesis model was proposed, which works using the encoder output, image captions, and so on^33,34^. The model under study works by receiving latent vectors from the previous language model, named the TBERT BASE CASE model. The latent output vectors are fed into the generator network, the noise function, and the preprocessed image feature vectors. Similarly, the output of the generator network is fed into the discriminator along with the generator noise function.

**Figure 4:**
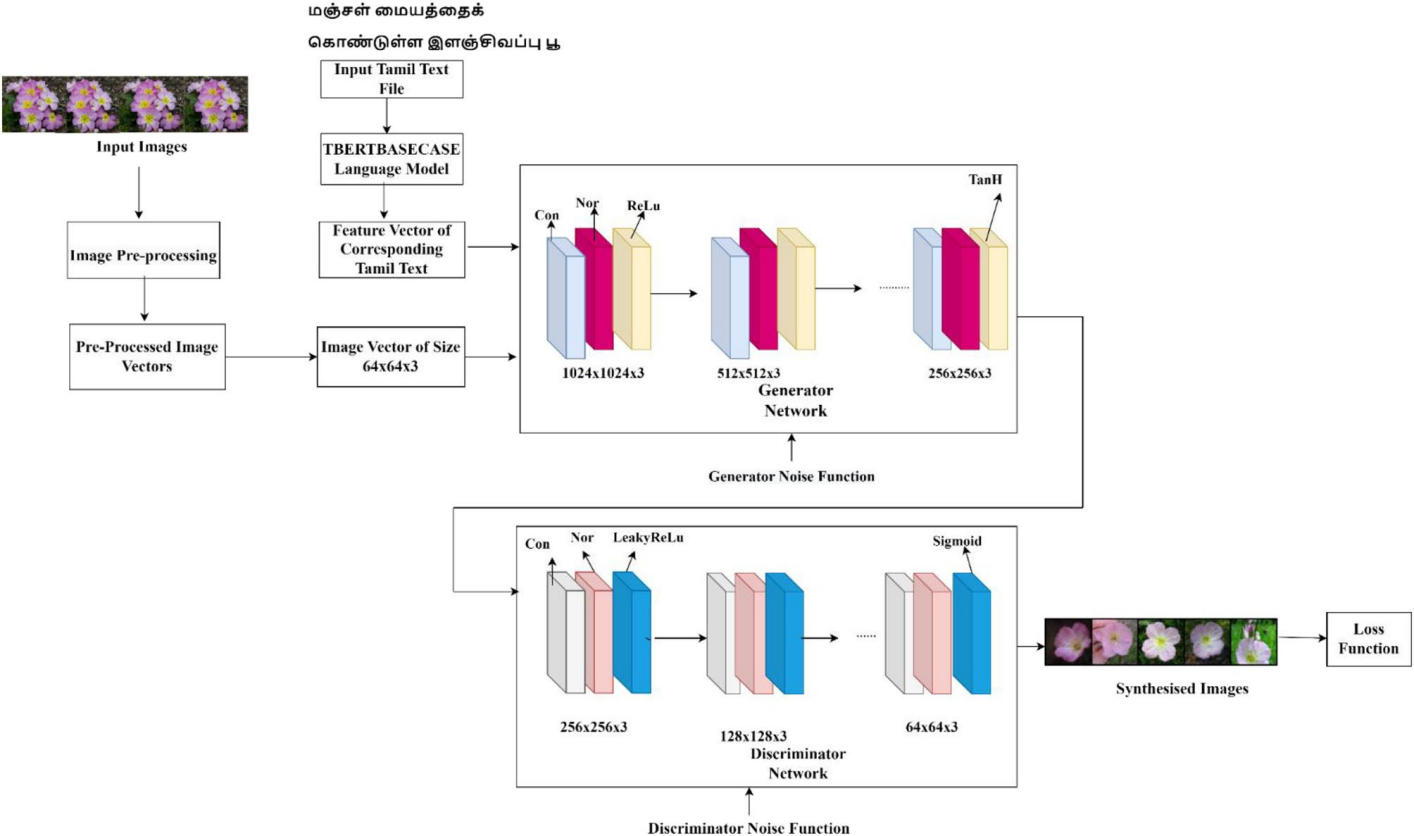
BASEGAN image synthesis network.

The two noise functions sent along with the text feature vectors are MINIMAX and Adversarial loss function^35^. The architecture is represented in Fig. 4.

The BASEGAN model has input image vectors of the image as I and text feature vectors as $\varphi ($ t). The image vectors are transformed to tensor values of size ${I}_{x}$ × ${I}_{y}$ × 3 which is the size of the image vector. The generic objective function of a GAN network s represented as Eq. (5),

5 $$minmaxGD\left(G,D\right)={E}_{x\sim\,pdata}[loglog (D\left(x\right)] +{E}_{z\sim\,pz}[loglog\left(1-D\left(G\left(z\right)\right)\right)]$$

The noise function *N*(0, 1) is supplied as a random value to the generator *G*, and the discriminator *D.* Let $\varphi ($t), the text vector, and $I$ be the corresponding image vector. The GAN model is trained in a fashion where the generator maximizes the loss ${L}_{D}$ of the discriminator and minimizes the generator loss ${L}_{G}$. The minimizing and maximizing equation is given by,

6 $${L}_{D}={E}_{\left(I,t\right)\sim pdata}[loglogD(I, \varphi \left(t\right))]+ {E}_{z}\sim pz[log(1-D\left(G\left(z\right), \varphi \left(t\right)\right))]$$

7 $${L}_{G}={-E}_{z\sim pz}[log(D\left(G\left(z\right), \varphi \left(t\right)\right)]$$

where $G\left(Z\right)$—Noise Function of Generator. $D\left(x\right)$—Discriminators probabilistic value that real instance is real. ${E}_{x\sim pdata}$-Expected overall real data. ${L}_{D}$—Discriminator Loss Function. ${L}_{G}$—Generator Loss Function. ${E}_{\left(I,t\right)\sim pdata}$—Expected over-all real data(image and text vectors). ${E}_{z\sim pz}$—Expected overall random inputs to the generator $\text{(input image and text vectors)}$

Equation (6 and 7) represents the loss function of a discriminator in maximizing and minimizing its objective function. $pdata$ refers to the distribution of the feature vector over the naturalistic image, and $pz$ refers to the data distribution over the latent space concerning the discriminator. In Eq. (6)The first term denotes the expectation over the logarithm of the discriminator’s output when given real samples x. The discriminator aims to maximize this term, correctly classifying real samples as close to 1. The second term represents the expectation over the logarithm of the discriminator’s output when given generated samples G(z). Here, z represents the random noise vector sampled from a prior distribution. The discriminator aims to minimize this term, classifying generated samples as close to 0.Eq. (7) represents the generator loss function.The expectation over the logarithm of the generated samples G(z). z is the random noise vector sampled prior distribution. The generator maximises this term to make the discriminator classify the generated samples as real.

### Hybrid super resolution GAN (HSR GAN)

**Figure 5:**
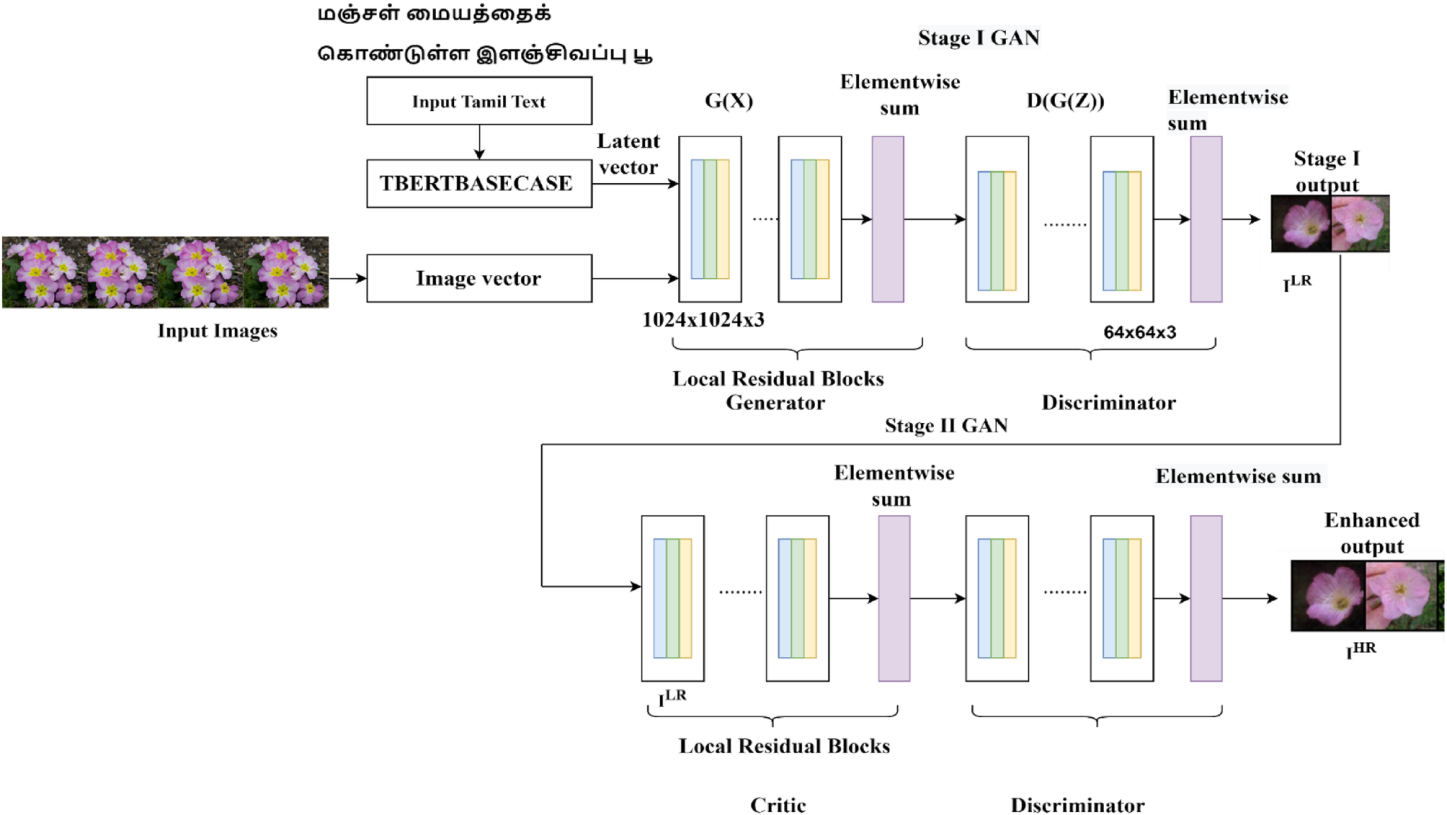
The architecture of Hybrid Super Resolution GAN (HSR GAN) model.

The Hybrid Super Resolution GAN (HSR GAN) works similarly to a two-stage GAN model. The super-resolution GANs^36^ proved to be a better image synthesis model in various applications than the conventional GAN model. The two-stage model works by super-resoluting the synthesised images from stage I. Many applications work with single-stage GAN network, but still it lacks minute information which are essential parts of a synthesised image. The model works with residual blocks. The Super Resolution GAN^37^ has two-stage models which work similarly to the Base GAN model considered in stage I, and the output is fed into stage II for improving resolution. The model works like residual blocks^38^ that have splitting, transforming, and aggregating functions. The vector representation of the image vectors is provided in the form of latent vectors where the essential feature vectors from latent space^39^ can be pointed to generate images by the generator. The image size of 1024 × 1024 is fed as an input to the generator of stage I, along with word embeddings from the TBERT BASECASE language model. The model architecture of the two-stage Hybrid Super Resolution GAN network is described in Fig. 5.

The Stage II GAN receives output images from stage I to further improve its resolution. The hybrid super-resolution GAN works. The Wasserstein loss function is employed in both the generator and discriminator^40^. The Wasserstein function’s significant advantage is bringing down the difference between original and fake images generated^41^. The two components involved are the natural distribution of values ${P}_{Real}$ and ${P}_{Generated}$, the distribution of values over the images generated. In mathematical form, it is the minimum distance to follow the Earth mover’s distance calculated by considering the transfer plan in which data is converted to authentic and generated images. The gain of experiencing the Wasserstein loss function is to create a large gap across real and fake images generated, which intends to solve the problem of understanding the realness of the image that is being generated. Moreover, it also helps in stabilizing the model during training of the model. The Wasserstein loss function creates a notion for the discriminator as a critic where it tries to bring out important gradient information everywhere in the training model.

The representation of HSRGAN is as follows: ${I}^{HR}$ refers to the high resolution of the image being generated and ${I}^{LR}$ being the representation of the low-resolution image generated by the stage I stage network. The objective function is represented in Eq. (8)

8 $$minmax{E}_{{I}^{HR}}\left(G,D\right)={E}_{x\sim pdata}\left[log\mathrm{log}D \left({I}^{HR}\right)\right]+{E}_{{I}^{LR}\sim p{I}^{LR}}\left[loglog\left(1-D\left(G\left({I}^{LR}\right)\right)\right)\right]$$

The loss function corresponding to stage II is Wasserstein loss which is represented as,

9 $${Loss}_{WDG}=E\left(C\left({x}_{real}\right)\right)-E(C(G\left(z\right))$$

From Eq. (9), it is clear that the Wasserstein loss function aims to evaluate the critic value of the real image distribution and the fake images generated. C denotes the critic value of the loss function. The critic value is any number, and the discriminator output is determined as critic output which depends on the Lipschitz constant, which is depicted in Eqs. (10 and 11)

10 $$c=1-Lipschitz\,Continuous$$

11 $${Distance}_{EMD}={\sum }_{i=1}^{m}{\sum }_{j=1}^{n}{[x}_{Real}G\left(z\right)]ij$$

where ${\mathrm{I}}^{\mathrm{LR}}$—being the representation of low-resolution image that is generated by stage-I stage network. ${\mathrm{I}}^{\mathrm{HR}}$—refers to the high resolution of the image that is being generated by the stage-II network. ${E}_{{I}^{HR}}$—$\text{Expected overall Real Data of}$ high-resolution image generated by stage-II network. ${E}_{{I}^{LR}\sim p{I}^{LR}}$—Expected overall Real Data of low-resolution image generated by stage I network. $D\left(G\left({I}^{LR}\right)\right)$—Probabilistic value that a fake instance of a low-resolution image is actual. C—The critic value is any number, and the discriminator output is determined as the critic output. ${Distance}_{EMD}$—Earth mover distance between real and fake distribution.

## Results and discussions

The model is evaluated using the F1 score, FID, and IS. The IS calculates the Kullback–Leibler (KL) divergence between conditional distribution p(y|x) and marginal distribution p(y), the InceptionScore.F1 score calculates the Precision and Recall values of the synthesised images. The resolution of the images tends to be of lower grade, and it can be enhanced over a two-stage GAN which is explained in the forthcoming model. The image obtained as resultant is convincing, but still it lacks the high-resolution features. It paves the way for enhancing the output from a BASEGAN model using the HSRGAN model. The testing and training set is represented in Table 2.

**Table 2:** Dataset of CUB-200 and Oxford-102.

Datasets	Train	Test
CUB -200	9414	2374
Oxford-102	7034	1155

**Figure 6:**
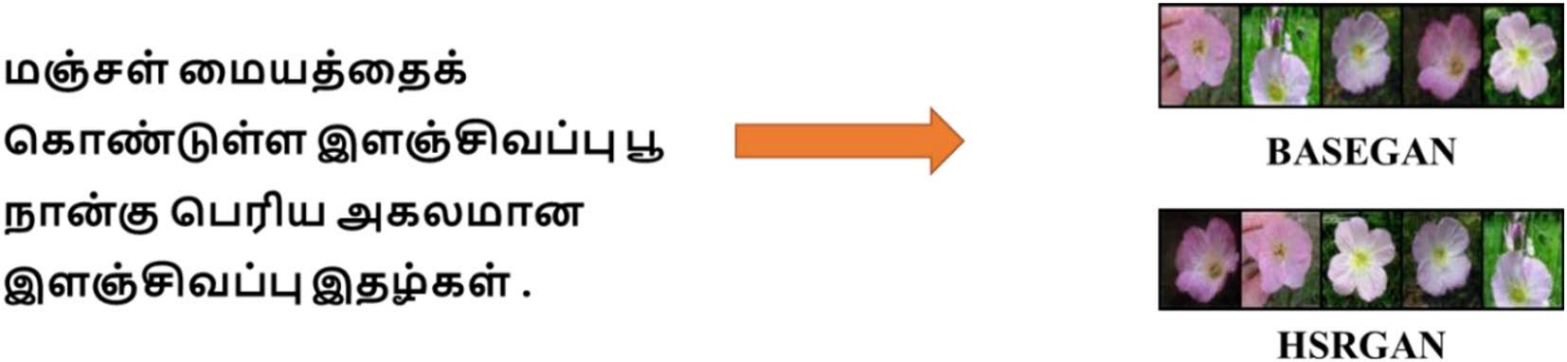
Generated Images Using BASEGAN and HSRGAN Network on Oxford-102 Dataset.

**Figure 7:**
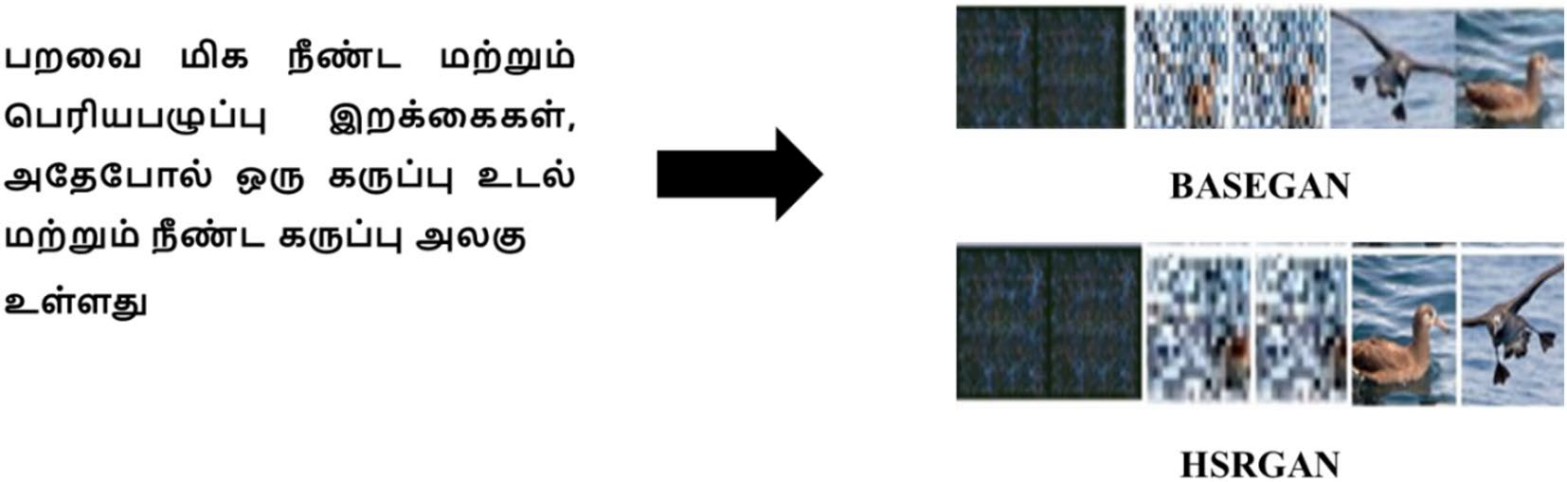
Generated Images Using BASEGAN and HSRGAN Network on CUB Dataset.

**Figure 8:**
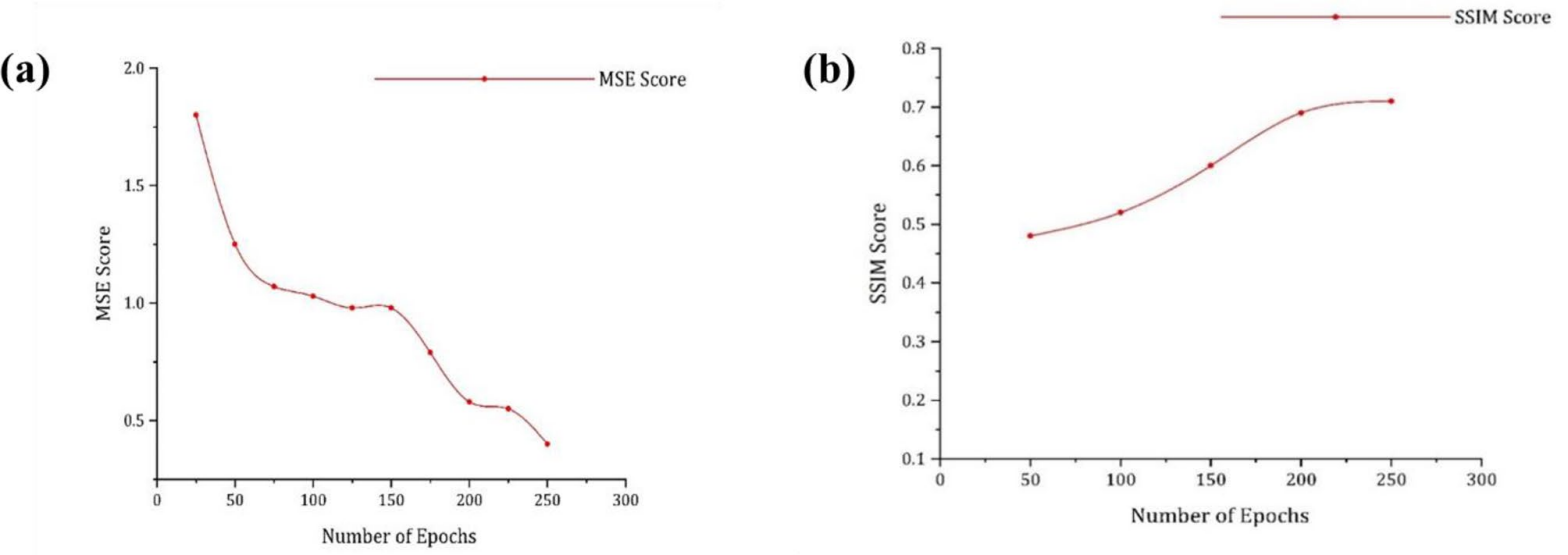
(**a**) MSE score for generated image quality Using HSRGAN (**b**) SSIM similarity index for generated image quality Using HSRGAN.

Figures 6 and 7 shows the images generated as a result of BASEGAN and resolution improvement through HSRGAN; from Fig. 8a,b, resolution improvement to a better level which can be dealt with by the Mean Squared Error (MSE) and Structural Similarity Index measure (SSIM) across epochs of training and the values on SSIM. When MSE is considered to evaluate the minimal similitude of the natural and fake images, the score more resembles the images because it provides a better reconstruction of the images. In initial epochs, the MSE value is at a higher rate, gradually decreasing, which helps reconstruct images. MSE figures out the deviations of the estimated features of images generated in terms of the square of the difference of pixel values. In the given case, there is no discrepancy between the images of stage I and those generated from stage II in terms of similar pixel values, which proves that there is only enhancement of features at two levels.

Similarly, SSIM is also used in measuring perceived image quality. It measures the brightness; the contrast of the image, and it also symbolizes that the higher the value of SSIM then, the quality of the image generated. The formulation of MSE and SSIM Eqs. (12 and 13).

12 $$MSE = \frac{1}{PQ }\mathop \sum \limits_{n = 0}^{P} \mathop \sum \limits_{m = 1}^{Q} [I^{HR} \left( {n,m} \right) - I^{LR} \left( {n,m} \right)]^{2}$$

13 $$SSIM (I^{HR} ,I^{LR} ) = \frac{{\left( {2\mu_{HR} \mu_{LR} + C_{1 } } \right)\left( {2\sigma_{HR} \sigma_{LR} + C_{2} } \right)}}{{\left( {\mu_{HR}^{2} \mu_{LR}^{2} + C_{1} } \right)\left( {\sigma_{HR}^{2} \sigma_{LR}^{2} + C_{2} } \right)}}$$

where μ_HR_ and μ_LR_ are the means, σ_HR_ and σ_LR_ are the standard deviations, and σ_HRLR_ is the cross-covariance for generated images x and real image input sequentially.

The Inception Score is represented in Eq. (14) as follows,

14 $$IS = {\text{exp}}\left( {E_{x} D_{KL} \left( {P(y|x)|{|}P\left( y \right)} \right)} \right)$$

x is a produced imagery analysis, and y is a pre-trained Inception v3 network image label^40^. Higher IS produces higher-quality images that are classified. Another Fréchet distance metric is FID. FID predicts feature space pictures using the pre-trained Inception v3 network. Equation (15) shows the FID score.

15 $$FID=||{\mu }_{x}-{\mu }_{z}{||}^{2}+Trace\left({\sum }_{x}+{\sum }_{z}-2*\sqrt{{\sum }_{x}{\sum }_{y}}\right)$$

Where, mean and covariance of the real and synthesised image. In contrast to IS, FID on the minimum value shows more realistic images and the distributions are alike. The F1 measure provides a combined way to represent precision and recall that captures both properties. Equation (16 ) represents the F1 measure,

16 $$F1\,Measure=\frac{2*Precision*Recall}{Precision+Recall}$$

GAN proved to be of outstanding practice in image synthesis applications. The model in hand for image synthesis for Tamil has two levels of performance the Base GAN model and the HSRGAN model. The primary concept of the proposed work is that it is the novel idea brought into the limelight for the Tamil language, which is at the development stage. It has a long way to explore. The proposed architecture can be a starting point for further enhancement in Tamil language that supports various applications in the regional language. At the initial stage, the language model aims to generate vector representations for Tamil text input. On the rear end, the GAN models synthesize images corresponding to the text vectors. The strength of the model lies in how far it can generate images.

Applications in English language have been at par, and various models have been proposed. The model proposed by the authors works for text-to-image and reversal in a vice-versa format^42^. The model employed by the authors is the Image-Text-Image model using the GAN model. The method can be used for any calligraphy. The CASIA-HWDB dataset and Chinese database of handwritten characters were tested with the model. Transfer models gave them convincible accuracy over the characters’^43^. Compared to English, much has been done. Pre-trained English text vectors like WordtoVec, Skip thinking vectors, Glove embeddings, and others can preprocess text. The GAN model matches AttnGAN. GAN uses image and text encoders. Using a common MS-COCO dataset, bidirectional LSTMs processed text and data from white, top, pillows, and table groups. Evaluation scored using BLUE-1, BLUE-2, 3, and 4.

However, past studies generated photos with English text descriptions, which required much technology^44^. The methodology proposed uses challenging and relevant Tamil text descriptions. The primary model is the work brought to lime light in the Tamil language. Previous work in this research is done for the English language. So, a comparative performance evaluation has been done with embedding vectors generated by the TBERTBASECASE language model along with the pre-trained GAN network like AttnGAN, and Stack +  + GAN. The proposed architecture is evaluated with the existing architecture, and its performance is assessed.

**Figure 9:**
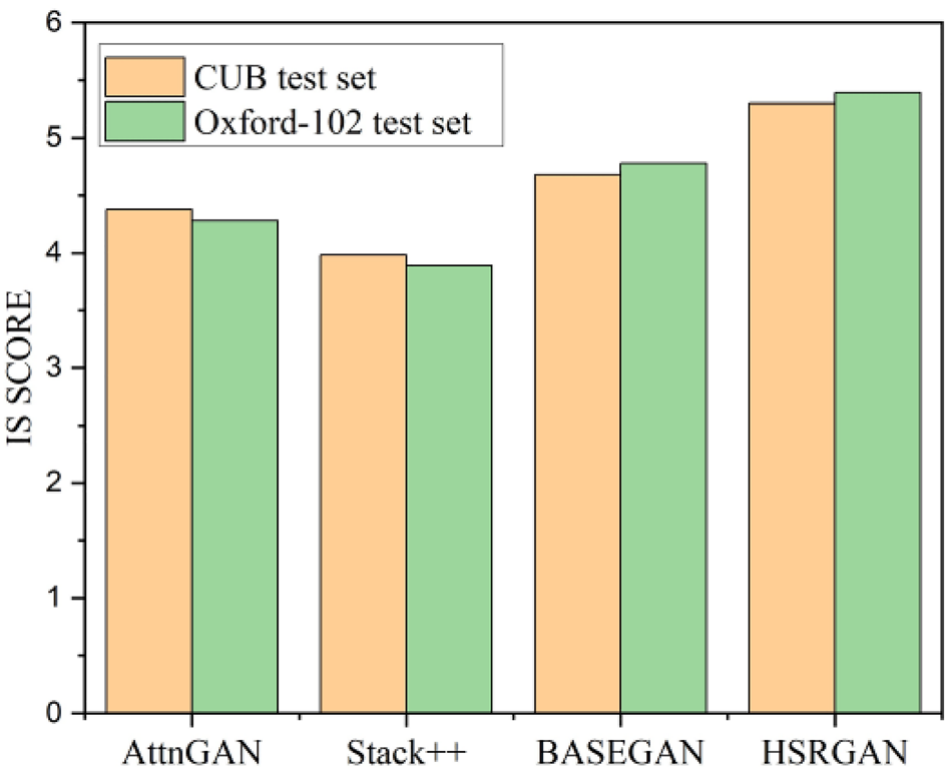
Inception Score evaluation.

**Figure 10:**
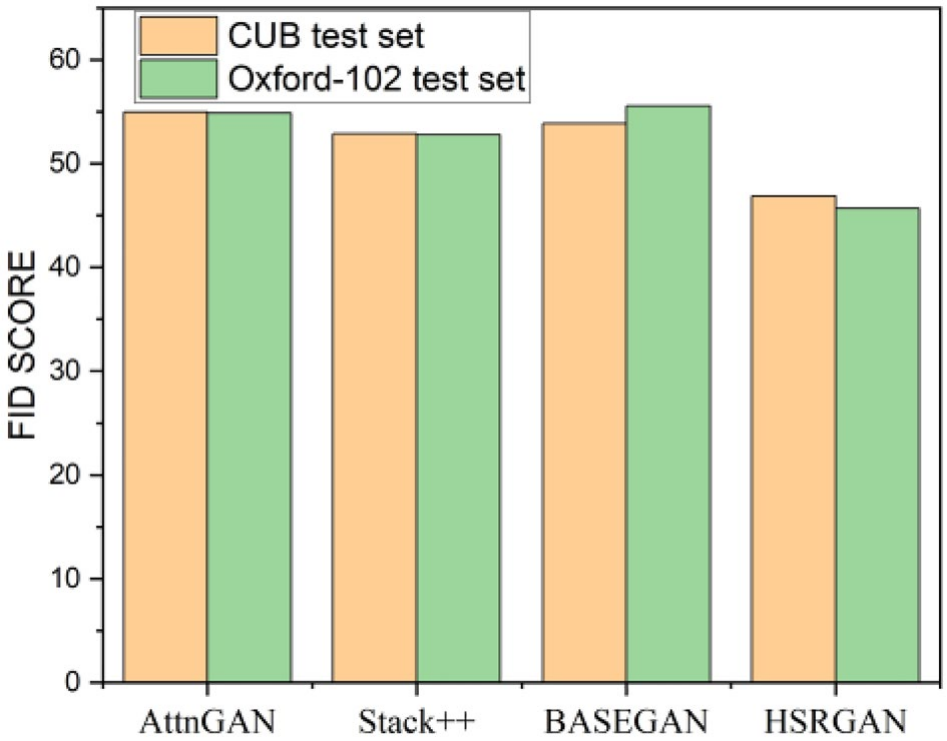
FID score performance estimation.

**Figure 11:**
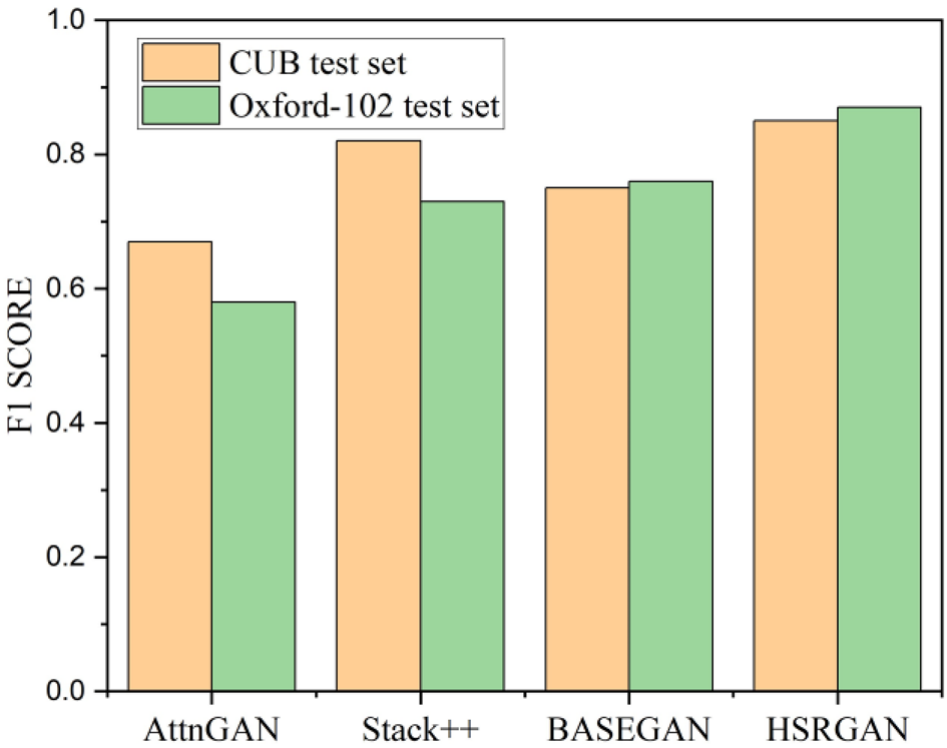
F1-Score valuation.

Figure 9 shows the valuation of IS metric on CUB-200 and Oxford-102 test sets. Among the proposed models, HSRGAN outperforms the BASEGAN and pre-trained GAN architecture. BASEGAN is comparatively better, along with the other pre-trained models. Figure 10 portrays the performance concerning the FID score. Here too, the proposed HSRGAN outperforms the trained architecture. The scores are convincing and satisfactory. Next on the run, Fig. 11 proves the performance of the architectures for the F1 score. Among the provided models, the projected work proves to be better in evaluation. Despite Tamil text processing’s complexity, the proposed model outcome matches StackGAN +  + . Thus, quantitative comparisons indicate that a persuasible performance in synthesising realistic and high-resolution images conditioned with Tamil text has been accomplished.

## Conclusion

Synthesis of images for text is an intriguing stream of research in computer vision. Image synthesis for Tamil text is critical since it has a rich morphology. TBERTBASECASE language and BASEGAN models are in the early stages of combining the Tamil language with the well-known GAN model. However, still, the performance of the basic architecture is convincible. It needs an improvement which is incorporated using a super-resolution GAN architecture. The BASEGAN model is enhanced by using a two-stage GAN model named Hybrid Super Resolution GAN(HSRGAN) to revamp the resolution of the images further. The model can be further improved using enhanced patterns of high-resolution GAN models and autoregressive models.

## Data Availability

The datasets generated and analysed during the current study are not publicly available since it is related to the doctoral thesis. After the submission of the research work, it would be provided. Nevertheless, are available from the corresponding author on reasonable request.
